# Treatment of Bloodstream Infections Due to Gram-Negative Bacteria with Difficult-to-Treat Resistance

**DOI:** 10.3390/antibiotics9090632

**Published:** 2020-09-22

**Authors:** Matteo Bassetti, Antonio Vena, Chiara Sepulcri, Daniele Roberto Giacobbe, Maddalena Peghin

**Affiliations:** 1 Clinica Malattie Infettive, Ospedale Policlinico San Martino—IRCCS, 16132 Genoa, Italy; matteo.bassetti@unige.it (M.B.); chiara.sepulcri@gmail.com (C.S.); daniele.roberto.giacobbe@gmail.com (D.R.G.); 2 Department of Health Sciences, University of Genoa, 16132 Genoa, Italy; 3 Department of Medicine, Infectious Diseases Clinic, University of Udine and Azienda Sanitaria Universitaria Integrata, 33100 Udine, Italy; maddalena.peghin@gmail.com

**Keywords:** Gram-negative bacteria, difficult to treat bacteria, *Enterobacteriales*, *Pseudomonas aeruginosa*, *Acinetobacter baumannii*

## Abstract

The rising incidence of bloodstream infections (BSI) due to Gram-negative bacteria (GNB) with difficult-to-treat resistance (DTR) has been recognized as a global emergency. The aim of this review is to provide a comprehensive assessment of the mechanisms of antibiotic resistance, epidemiology and treatment options for BSI caused by GNB with DTR, namely extended-spectrum Beta-lactamase-producing *Enterobacteriales*; carbapenem-resistant *Enterobacteriales*; DTR *Pseudomonas aeruginosa*; and DTR *Acinetobacter baumannii*.

## 1. Introduction

In the last two decades, antibiotic resistance has grown at such a pace, and spread so widely, that it has been recognized as a global emergency.

Among the pathogens causing concern, Gram-negative bacteria (GNB) have raised the most attention, due to the rapid increase in their association with morbidity and mortality all over the world [1]. Bloodstream infections (BSI), present in 40% of cases of community-acquired and hospital-acquired sepsis and septic shock, are associated with poor outcomes, especially with delayed adequate antimicrobial therapy and source control [2]. GNB BSI isolates represent an usufeul surveillance target for the monitoring of resistance, as cultures from sterile sites avoid the problem of confounding colonizing agents.

In 2008, the US and European Centers for Disease Control and Prevention (CDC and ECDC) proposed a classification of resistance based on the phenotypic profiles of resistance to antimicrobials. Three resistance phenotypes were defined: multidrug resistance (MDR), defined as non-susceptibility to ≥1 agents in ≥3 antimicrobial categories; extensive drug resistance (XDR), susceptibility limited to ≤2 categories; and pan-drug resistance (PDR), non-susceptibility to all agents in all antimicrobial categories [3]. This classification has been extensively used in the literature, but its usefulness in clinical practice has been recently questioned [4]. Despite their advantages for epidemiological studies, these definitions share several limitations, as they do not differentiate between or prioritize antibiotic classes according to their efficacy or toxicity and mean that very broad classes of antibiotics must be tested. Above all, the problem is that their use does not correlate with clinical outcomes [5].

In 2018, Kadri et al. proposed a new term for the multidrug resistance profile of GNB, namely difficult-to-treat resistance (DTR), which these authors defined as any GNB-BSI isolates demonstrating an intermediate or resistant phenotype to all first line agents in the carbapenem, beta-lactam, and fluoroquinolone categories [5]. DTR means non-susceptibility to all first-line, high-efficacy, low-toxicity agents and focuses on the effect of resistance on treatment decisions and clinical outcomes. This new definition has already been accepted by several authors, and its association with clinical outcomes is currently under evaluation. It has received both praise and criticism, since with the introduction of new antibiotics, the concept of DTR will evolve in keeping with the assessment of what constitutes first-line treatment [6,7,8].

The rising incidence of BSI due to GNB with DTR is a matter of serious concern, both in the community and in the hospital setting [2,9]. The aim of this review is to provide a comprehensive assessment of the currently available and emerging treatment options for BSI caused by GNB with DTR, namely extended-spectrum Beta-lactamase (ESBL)-producing *Enterobacteriales*; carbapenem-resistant *Enterobacteriales* (CRE); DTR *Pseudomonas aeruginosa*; and DTR *Acinetobacter baumannii*.

## 2. DTR Pathogens: Mechanisms of Antibiotic Resistance and Epidemiology

### 2.1. Enterobacteriales

The bacterial mechanisms most commonly involved in antibiotic resistance are the modification of the antibiotic molecule, decreased antibiotic penetration and efflux, changes in the antibiotic target site, and global cell adaptation. DTR *Enterobacteriales* mechanisms of resistance mainly rely on the production of beta-lactamases, enzymes that catalyze the destruction of the amine bond of beta-lactam antibiotics in the periplasmic space. These enzymes have been extensively studied, and are normally classified using the Ambler classification. The Ambler scheme divides β-lactamases into four major classes (A to D), according to their amino acid similarity. In the Ambler scheme, β-lactamases of classes A, C and D are classified as serine β-lactamases (Table 1).

### 2.2. ESBL-Producing Enterobacteriales

ESBLs are a group of beta-lactamases that confer resistance to penicillins, third generation cephalosporins (the hallmark characteristic) and monobactams, while they are inactive against carbapenems. The main ones (TEM-, SHV- and CTX-M-type enzymes) fall under Ambler class A and are plasmid-encoded, while other enzymes, such as AmpC and Oxa-11, are included in classes C and D respectively. Since their discovery in the 1960s, their prevalence has been rising worldwide. Although global data on the prevalence of specific mechanism of resistance are still lacking, the 2018 ECDC surveillance report showed that the prevalence of *E.coli* resistant to third generation cephalosporins in the EU/EEA region was 15.1%, while that of *K. pneumoniae* reached 31.7%, with peaks of >50% in Italy, Portugal and Eastern Europe. ESBL-producing *Enterobacteriales* are frequently encountered as colonizers or causes of infections, both in the community and in the hospital setting [10].

### 2.3. Carbapenem-Resistant Enterobacteriales

Resistance to carbapenems is caused by the production of carbapenemases, which fall under more than one Ambler class. Less frequently, carbapenem resistance stems from deficient outer-membrane protein expression. In Ambler class A, the main enzymes are KPC (*Klebsiella pneumoniae* carbapenemase), which is plasmidic, and SME *(Serratia marcescens* enzyme), which is chromosomally transmitted; these enzymes are not inhibited by aztreonam, which they hydrolyze [11] (Table 1).

Enzymes classified in Ambler class B are all carbapenemases belonging to the metallo beta-lactamase (MBL) group, due to their beta-lactamase activity through a metal ion (usually zinc) as cofactor. There are three main MBLs: imipenemase (IMP), Verona integron-encoded metallo beta-lactamase (VIM) and New Delhi metallo beta-lactamase (NDM). Encoded by plasmids or other mobile genetic elements, these MBLs are spreading rapidly all over the world and are now the mechanism of resistance of greatest concern; they are frequently associated with the expression of additional genes conferring resistance to other antimicrobial drug classes such as fluoroquinolones and aminoglycosides, and thus narrow the spectrum of therapeutic options. They are not inhibited by old beta-lactamases inhibitors (clavulanic acid or tazobactam) or by new ones (avibactam, relebactam or vaborbactam), but they remain susceptible to aztreonam [12] (Table 1). The ability of these enzymes to spread rapidly is demonstrated by their capacity to cause outbreaks in non-endemic regions, as recently occurred in the outbreak of NDM-producing *K. pneumoniae* in Tuscany [13,14].

Class D enzymes pertain to the OXA-48 class, and have an unusual mechanism of resistance; they are penicillinases (particularly oxacillinases) and carbapenamases, but they are susceptible to third generation cephalosporins, although they frequently co-produce as ESBL enzymes and thus confer resistance to all beta-lactams [15]. They were first described in a *K. pneumoniae* isolate in Turkey, but can be now found in all *Enterobacteriales* (especially *E. coli*) throughout the world, particularly in North Africa and the Middle East, where they are endemic [16].

From the epidemiological point of view, data from the latest ECDC report showed that carbapenem resistance remained rare (<1%), although several countries reported high percentages (above 10% or even 25%) of carbapenem-resistant *K. pneumoniae*, including Italy, France, Spain, Portugal and most Eastern European countries. Greece reported the highest levels of resistance with a prevalence of 50% [10].

Carbapenem-resistant *Enterobacteriales* (CRE) enzymes (except for OXA-48) are mainly encountered in the hospital setting in patients receiving prolonged antibiotic therapy, carrying indwelling devices, or undergoing long-term hospitalization or ICU admission [17].

### 2.4. Pseudomonas aeruginosa

*P. aeruginosa* deploys almost all the resistance mechanisms available to bacteria to protect itself from the action of antibiotics, and is one of the most difficult organisms to treat. Its preferred mechanism of resistance involves the efflux pumps that remove beta-lactams and fluoroquinolones, as well as many dyes and detergents. Other mechanisms include the production of inducible AmpC enzyme, the loss of impermeability due to loss of porin OprD, which is particularly associated with a reduced susceptibility to carbapenems, and acquired enzymatic mechanisms of resistance, such as OXA, ESBL enzymes and MBL enzymes, which have led to the spread of multidrug resistance [18]. In 2018, about 19% of *P. aeruginosa* isolates in the EU/EEA were resistant to fluoroquinolones, 18.3% to piperacillin/tazobactam, 17.2% to carbapenems and 14.1% to ceftazidime. Combined resistances to two, three or five antimicrobial classes were 7.6%, 4.1% and 4.1% respectively—rates that indicate the need for new drugs active against these DTR strains [10] (Table 1).

### 2.5. Acinetobacter baumannii Complex

Like *P. aeruginosa,* species of the *A. baumannii complex* have developed multiple mechanisms of resistance to many classes of antibiotics, and have often become DTR organisms in the context of healthcare-associated infections. The principal mechanisms of resistance are the production of chromosomally encoded AmpC cephalosporinases (as well as other β-lactamases of the TEM, CTX-M SHV type). However, the most recent and worrisome mechanism of resistance is the production of carbapenemases (both serine and MBLs), which, in the case of the *A. baumannii* complex, are mainly OXA-23 for the serine type and IMPs and VIMs for the MBL class. Other typical mechanisms of resistance are changes in the porins, production of aminoglycoside-modifying enzymes, and efflux pumps [19]. The prevalence of antibiotic resistance among *A. baumannii* complex isolates is a matter of particular concern. In 2018, in the EU/EEA, 31.9% of isolates were reported to be resistant to carbapenems; the same percentage were resistant to aminoglycosides and an even higher percentage to fluoroquinolones (36.2%); 28.8% of isolates presented combined resistance to all these three classes, a situation that severely jeopardized treatment options [10] (Table 1).

## 3. Novel Treatment Options

### 3.1. Ceftolozane/Tazobactam

Ceftolozane/tazobactam (C/T) is a novel β-lactam/β-lactamase inhibitor (BL/BLI), which exhibits excellent in vitro activity against *Pseudomonas aeruginosa*, including drug-resistant strains, and other *Enterobacteriales* including most ESBL. This new drug is currently EMA and FDA approved for the treatment of complicated intra-abdominal infections (cIAI) (in combination with metronidazole) and complicated urinary tract infections (cUTI) on the strength of the results of the ASPECT-cIAI and ASPECT-cUTI trials [20,21]. Recently, C/T has also been approved for hospital-acquired pneumonia (HAP) and ventilator-associated pneumonia (VAP), since high-dose ceftolozane/tazobactam (3 g q 8 h) achieved non-inferiority vs. meropenem in the ASPECT-nosocomial pneumonia (NP) trial [22] (Table 2 and Table 3).

### 3.2. ESBL

Carbapenems remain the treatment of choice for severe ESBL infections [23]. The role of carbapenem-sparing regimens for ESBL infections in order to reduce selective antimicrobial pressure is unclear.

The data available on the use of ceftolozane–tazobactam for ESBL pathogens are limited, and are mainly extrapolated from pivotal clinical trials. Regarding ESBL producers, the activity of C/T has been shown to be superior overall to that of piperacillin/tazobactam, but meropenem showed better in vitro activity against *Enterobacteriales*, especially against ESBL-producing strains. Nonetheless, when patients with ESBL infections treated with ceftolozane–tazobactam were compared to those receiving meropenem, there were no differences in terms of clinical outcomes between treatment groups [22,24,25].

A recent Italian multicenter retrospective study reported the largest clinical experience regarding the use of C/T therapy for the treatment of serious ESBL-producing *Enterobacteriales* infections in daily clinical practice (NP 31.7%, acute bacterial skin and skin-structure infection [SSTI] 20.8%, cUTI 13.9%, cIAI 12.9%, bone infection 8.9% and primary bacteremia 5.9%), and showed C/T to be a valid option in empiric and/or targeted therapy, with favorable clinical outcomes in 128/153 (83.7%). Interestingly, the risk of clinical failure was associated with standard-dose C/T therapy in septic patients receiving continuous renal replacement therapy (CRRT) [26].

The use of ceftolozane/tazobactam as a carbapenem-sparing strategy for infections due to ESBL-producing *Enterobacteriales* has also been proposed, and may be useful and cost-effective in selected scenarios depending on local antibiotic stewardship decisions [27]. More evidence is needed to define the therapeutic role of ceftolozane/tazobactam for ESBL infections [28].

AmpC producers are susceptible to C/T to different degrees, depending on the bacterial species and enzyme types in question. Although there is no relevant clinical information, the activity of C/T against *Enterobacteriales* with copious AmpC enzyme is variable, and many *Enterobacter* spp. with de-repressed AmpC are resistant to this antibiotic, which should not be used in infections due to AmpC-producing species [29,30].

### 3.3. DTR Pseudomonas aeruginosa

C/T can be considered the most potent agent against *DTR P. aeruginosa*, due to its activity against common resistance mechanisms showed by *P. aeruginosa*, including loss of outer membrane porin (OprD), chromosomal AmpC, and up-regulation of efflux pumps (MexXY, MexAB), the exception being the carbapenamase-producing strains. A large multicenter study recording the clinical experience with this agent at 22 Italian centers underlined its efficacy in treating infections caused by *P. aeruginosa*, regardless of the site of origin [31] C/T is often used as the backbone of combination therapy involving dual treatment active against *P. aeruginosa,* for example in HAP/VAP or in high-mortality risk patients. In this context, it is often associated with aminoglycosides, colistin, fosfomycin. Possible synergisms between ceftolozane/tazobactam and other agents (including fosfomycin, colistin, and amikacin) in the treatment of *P. aeruginosa* are being studied in vitro and may lead to the development of new treatment options [32,33].

### 3.4. CRE and DTR Acinetobacter

Ceftolozane/tazobactam is inactive against carbapenemase-producing isolates, since it lacks activity against CPE (MIC50/90, 32/>32 mg/L); Moreover, C/T shows no activity against metallo-β-lactamases, *K. pneumoniae* carbapenemases, or class D enzymes [34].

### 3.5. Ceftazidime/Avibactam

Ceftazidime/avibactam (CAZ-AVI) is a BL/BLI combination of an old cephalosporin (ceftazidime) with a new BLI (avibactam). This drug shows activity against ESBL, class C (e.g., AmpC) β-lactamases, class A (e.g., KPC) and class D (e.g., OXA) CRE. In addition, it has demonstrated activity against some DTR *P. aeruginosa* isolates (Table 2). CAZ-AVI was approved in 2015 by the FDA for complicated cUTI, cIAI, HAP and VAP in adult and pediatric populations. Its use has also been extended by the European Medicines Agency (EMA) to infections due to aerobic GNB in patients with limited treatment options. This BL/BLI combination has rapidly become one of the mainstays in the treatment of DTR-GNB infection, and its use in real-life clinical situations has demonstrated its value in the treatment of other clinical syndromes such as primary BSI [27].

In our opinion, CAZ-AVI should be also considered as a possible therapeutic option for the treatment of severe infections due to ESBL- or AmpC-producing species, as well as DTR *P. aeruginosa*. The merits of this choice should be weighed up against those of reserving it for carbapenem-resistant strains [35] (Table 2 and Table 3).

### 3.6. ESBL

According to in vitro studies results, some authors have proposed the use of CAZ-AVI as a potential carbapenem-sparing option for serious infections due to ESBL-producing *Enterobacteriales*. CAZ-AVI demonstrated high in vitro potency against clinical isolates of *Enterobacteriales*, since it was the most active against non-susceptible ceftazidime isolates of the comparator agents tested (97.7% susceptibility) [36], and its effect was shown to be unchanged regardless of the ESBL enzyme and species tested [24]. A post hoc analysis of phase 2 trials showed no differences in terms of efficacy between ceftazidime-avibactam and carbapenems for treatment of cUTI and cIAI due to ESBL *Enterobacteriales* and/or AmpC β-lactamases [37,38].

Post-marketing experience regarding the use of CAZ-AVI for infections due to ESBL-producing *Enterobacteriales* remains limited. A recent real-life retrospective multicenter study in 41 patients of the value of CAZ-AVI for the treatment of serious infections due to DTR GNB other than CRE found excellent clinical success for ESBL (100%, 4/4), surpassing even the results obtained in the early pivotal trials [39].

### 3.7. CRE

In real-life experience, high favorable response rates have been reported in patients with infections due to KPC-producing *Enterobacteriales* treated with ceftazidime-avibactam, with an overall success rate around 70% [40,41]. Among patients with KPC-KP BSI, 30-day mortality rates were significantly lower in the ceftazidime/avibactam group than in a matched group of patients receiving other treatments (36.5% versus 55.7%, *p* = 0.005) [42]. The reports of the emergence of resistance during therapy to this new combination due to blaKPC mutations are a matter of concern. Interestingly, pneumonia and renal replacement therapy have been associated with clinical failure and with the emergence of resistance during therapy among patients with CRE infections [43]. Despite few clinical data, ceftazidime/avibactam has shown promising results for salvage treatment of patients with severe infections due to OXA-48-producing *Enterobacteriales* and with limited therapeutic options, with no evidence of the emergence of resistance to ceftazidime/avibactam [44,45]. Ceftazidime/avibactam has no activity against metallo-β-lactamase-producing *Enterobacteriales* (Table 2), but previous clinical studies including patients with BSI due to MBL-producing *Enterobacteriales* have shown that combination therapy with CAZ-AVI plus aztreonam is an effective therapeutic option [14].

### 3.8. DTR P. aeruginosa

CAZ-AVI has shown high in vitro potency against clinical isolates of *P. aeruginosa* collected in European countries, including isolates that exhibit resistance to ceftazidime, meropenem and colistin and combined resistance to agents from multiple drug classes [46]. Overall, *P. aeruginosa* showed a resistance rate to CAZ-AVI ranging from 2.9 to 18% [47], and the activity of CAZ-AVI against DTR P. aeruginosa has proven to be slightly lower than that of C/T (i.e., with lower MICs against ESBL isolates) [48]. The use of CAZ-AVI in *P. aeruginosa* infections focuses on strains resistant to C/T by the production of ESBL or class A carbapenamases and to MBL-producing strains, in which combination treatment with aztreonam (+/−colistin) may be one of the few currently available therapeutic options [49].

### 3.9. DTR Acinetobacter

Resistance to CAZ-AVI exceeds 50% in *Acinetobacter baumannii*, and strains isolated from patients admitted to intensive care units (ICUs) have an even higher resistance rate, at 73.6%. In addition, DTR *Acinetobacter* spp. expressing *bla*_OXA-51_-like genes are completely resistant to CAZ-AVI [47].

## 4. Imipenem/Relebactam

Imipenem/relebactam is a combination of a carbapenem and a new potent non-β-lactam β-lactamase inhibitor (relebactam). Relebactam shows activity against class A and C beta-lactamases, but it is not active against MBL or class D carbapenemases. Imipenem/relebactam is currently approved by FDA for the treatment of cIAI and cUTI [50,51]. Imipenem/relabactam was compared to colistin plus imipenem in the RESTORE-IMI 1 trial, and proved to be an efficacious and well-tolerated treatment option for carbapenem-nonsusceptible infections [52]. Currently, imipenem/relebactam is being compared with piperacillin/tazobactam in RESTORE-IMI 2, a phase III clinical trial involving patients with NP, including VAP [53] (Table 2 and Table 3).

### 4.1. ESBL

The established therapeutic option for severe infections due to ESBL is a carbapenem without a β-lactamase inhibitor. Relebactam is a non-β-lactam compound that inhibits class A enzymes, including ESBL β-lactamases [54].

### 4.2. CRE

Imipenem/relebactam has a wide spectrum of activity against various phenotypes and genotypes of carbapenem-resistant strains, especially KPC-producing *Enterobacteriales*. Relebactam restores imipenem activity against carbapenem-resistant *Enterobacteriales* due to the impermeability caused by the loss of the porin OprD, in association with the expression of ESBL or AmpC β-lactamase. However, imipenem/relebactam shows no activity against MBL (VIM, IMP, or NDM) producing strains. However, relebactam may slightly enhance the activity of imipenem against OXA-type CRE.

### 4.3. DTR P. aeruginosa

Relebactam restores the activity of imipenem to class A carbapenemase-producing *P. aeruginosa*, and it is a poor substrate for efflux pumps. A recent study showed that it maintained activity in strains that became resistant to both ceftolozane/tazobactam and ceftazidime/avibactam, due to AmpC or OXA-10 mutations [55]. However, imipenem/relebactam is not active against IMP- or VIM-producing *P. aeruginosa*.

### 4.4. DTR Acinetobacter

Imipenem/relebactam has no activity against imipenem-resistant *A. baumannii*. Relebactam does not increase the number of isolates of *A. baumannii* susceptible to imipenem [56].

## 5. Meropenem/Vaborbactam

Meropenem/vaborbactam is a fixed-dose combination product of an old carbapenem (meropenem) with a cyclic boronic acid β-lactamase inhibitor (vaborbactam). The addition of vaborbactam to meropenem restores its activity against *Enterobacteriales* producing Ambler class A carbapenemases, especially against KPC-CRE. Vaborbactam does not inhibit Ambler class B or D carbapenemases [57]. Meropenem/vaborbactam is FDA- and EMA0approved for the treatment of cUT Ion the strength of the TANGO I trial, and is also EMA approved for the treatment of cIAIs and HAP including VAP [58]. The TANGO III trial for the treatment of patients with HAP and VAP is currently ongoing [59] (Table 2 and Table 3).

### 5.1. ESBL

The established therapeutic option for severe infections caused by ESBL is a carbapenem without a β-lactamase inhibitor. Vaborbactam is active against serine carbapenemase, and shows potent activity against class A EBSL, with CTX-M, TEM and SHV enzymes [60].

### 5.2. CRE

The TANGO II Phase 3 study supported the efficacy of M/V for the treatment of serious (cUTI, cIAI, HAP, VAP, and BSI) infections caused by CRE, since this combination was associated with improved clinical cure, decreased mortality, and fewer adverse events than the best available therapy in resistant pathogens [61], and presented better results in patients without prior antimicrobial failure [62]. Despite the paucity of data, M/V has promising efficacy in the setting of pneumonia and/or other severe KPC producing-CRE infections in a real-world setting [57,63]. In addition, the potential for resistance selection appears to be lower overall than that observed for CAZ/AVI and colistin. Some preclinical data suggest that M/V in association with aztreonam could have similar activity to CAZ/AVI plus aztreonam against CRE strains producing MBL provided that there are no OXA enzymes alongside MBL [64].

### 5.3. DTR Pseudomonas aeruginosa and DTR Acinetobacter

Vaborbactam shows no activity against Ambler class B or D carbapenemases, and it does not improve the activity of meropenem against DTR GNB. As a matter of fact, it has no effect on meropenem non-susceptible *A*. *baumannii* spp. producing OXA-type carbapenemases or on *P*. *aeruginosa*.

## 6. Cefiderocol

Cefiderocol is a novel siderophore cephalosporin which applies a “Trojan Horse” active transfer mechanism using the bacterial iron transport system [65]. It overcomes all classes of carbapenemases, porin channel mutations, and efflux pump overexpression. This combination of efficient cell entry and increased stability against various types of β-lactamases makes cefiderocol highly active in vitro against a variety of DTR GNB such as KPC and MBL-producing *Enterobacteriales*, *P. aeruginosa*-producing MBL and *A. baumannii*-producing OXA-type β-lactamase, showing activity against 96% of all tested clinical isolates [66,67].

Cefiderocol has recently (November 2019) been approved by the FDA for cUTI infections, with limited or no alternative treatment options after demonstrating its efficacy in the APEKS-cUTI study [68].

Although the results of further clinical trials investigating the efficacy of cefiderocol for treatment of severe infections with carbapenem-resistant GNB (CREDIBLE-CR) [69,70], NP (APEKS-NP) [71], and BSI (GAMECHANGER) [72] are pending, recent data suggest that cefiderocol may constitute a promising treatment option for infections caused by DTR GNB [73] (Table 2 and Table 3).

### 6.1. ESBL

Cefiderocol shows high stability against β-lactamases, including ESBL.

### 6.2. CRE

The minimum inhibitory concentrations (MICs) 90 of cefiderocol have been shown to be lower for *Enterobacteriales* than for nonfermenting GNB. On the basis of in vitro data, cefiderocol may become the first-line therapy for KPC-, NDM-, VIM-, IMP-producing isolates of *Enterobacteriales* [74]. Several studies have analyzed the activity of cefiderocol against OXA-48-type-producing *K. pneumoniae* isolates, and have reported MICs in the susceptible range (4 mg/L) [74]. Most studies have found KPC to be susceptible to cefiderocol, while some VIM-1-producing *E. cloacae* strains have previously been reported to be resistant.

### 6.3. DTR Pseudomonas aeruginosa

Cefiderocol has been reported to have potent in vitro activity against carbapenem-resistant *P. aeruginosa* [75]. It has already been successfully used as compassionate treatment in serious infections caused by DTR *P. aeruginosa* [76,77].

### 6.4. DTR Acinetobacter

Cefiderocol has potential for the treatment of *Acinetobacter baumannii*, in which therapeutic options are limited. It has shown potent in vitro activity against carbapenem-resistant *A. baumannii* [74]. On the basis of the available data, it is not possible to establish whether one type of oxacillinase has more activity against cefiderocol than another, but isolates of *A. baumannii* OXA-24/40 have shown resistance to this antimicrobial [74].

## 7. Other New Antibiotics

### 7.1. Aztreonam/Avibactam

Aztreonam is active against MBL-producing bacteria, but is hydrolyzed by Ambler class A and class C beta-lactamases (Table 1). Its combination with avibactam is able to inhibit cell wall synthesis in MBL-producing strains, despite the presence of other co-carried beta-lactamases or carbapenemases (Table 1 and Table 2) [78]. Aztreonam/avibactam showed a potent in vitro activity against ESBL, class C beta-lactamase, MBL, and KPC-producing strains, with a rate 10 times higher than aztreonam alone. However, its activity against *A. baumannii* or *P. aeruginosa* compared with aztreonam alone is limited [79]. Some phase III clinical trials are temporarily suspended, though others are ongoing [80,81] (Table 2).

### 7.2. Plazomicin

Plazomicin is a novel aminoglycoside, with enhanced activity against ESBL-producing, carbapenemase-producing (including metallo-β-lactamases) and aminoglycoside-resistant *Enterobacteriales*. It retains stability against several aminoglycoside-modifying enzymes; however, it is not active against many of the strains producing NDM carbapenemases. Plazomicin activity against *P. aeruginosa* and *A. baumannii* is overall comparable to existing aminoglycosides, but is not predictable [82]. This drug is currently FDA approved for the treatment of cUTI [83] and could be an excellent carbapenem-sparing alternative, particularly for cUTI caused by these DTR GNB. For more severe infections such as BSI caused by KPC *Enterobacteriales*, plazomicin may be administered in individual clinical scenarios if a combination therapy is considered [84] (Table 2 and Table 3).

### 7.3. Eravacycline

Eravacycline is a fluorocycline antibiotic in the tetracycline class with excellent in vitro activity against GNB, including CRE, carbapenem-resistant strains of *A. baumannii* and S. *maltophilia*, but not those of *P. aeruginosa*.

The FDA recently approved eravacycline for treatment of cIAIs in adults on the basis of the results of the IGNITE 1 and IGNITE 4 [85,86] studies, but its performance was shown to be inferior to levofloxacin and ertapenem in IGNITE 2 and 3 respectively for treating cUTI, presumably due to its suboptimal urinary pharmacokinetics [82]. The enthusiasm regarding this drug is tempered by the lack of clinical data on its use in pneumonia and bacteremia (Table 2 and Table 3).

### 7.4. Murepavadin

Murepavadin is one of a new class of antibiotics called outer membrane protein targeting antibiotics, which are highly active against DTR *P. aeruginosa,* as well as ceftolozane/tazobactam-resistant and colistin–resistant strains, but it is not active against other non-fermenting GNB or *Enterobacteriales*. Two clinical trials investigating the role of murepavadin in respiratory tract infections that were prematurely terminated due to safety concerns (i.e., high rates of renal failures in the murepavadin arm) and efficacy data for other types of infection, including BSI, are lacking (Table 2).

Below, we list several new promising antibiotics currently in development, whose clinical role will be determined by the breakpoints assigned to them, comparison studies with other investigational drugs, and the body of data on therapeutic outcome as they become known:

### 7.5. Cefepime/Zidebactam

Cefepime/zidebactam has demonstrated potent in vitro activity against DTR GNB, against *Enterobacteriales* (including ESBL AmpC, KPCs, and OXA-48-producing strains), *P. aeruginosa* isolates with multiple resistance mechanisms including upregulated efflux, loss of OprD porins, and AmpC overproduction and Acinetobacter [35,87] (Table 2).

### 7.6. Meropenem/Nacubactam

Meropenem/nacubactam is highly efficacious against most ESBL, KPC, MBLs, AmpC, and OXA-48 producing *Enterobacteriales*. In addition, the combination is active against *P. aeruginosa* with depressed AmpC, as well as against ESBL or AmpC-producing *Enterobacteriales* with porin loss. Nacubactam has the potential to extend meropenem activity to DTR, meropenem nonsusceptible and CAZ/AVI-resistant isolates. Meropenem/nacubactam has a similar activity to meropenem alone against *Pseudomonas* spp. and *Acinetobacter* spp. [56] (Table 2).

### 7.7. Ceftaroline/Avibactam

The combination of avibactam with ceftaroline expands its antimicrobial spectrum covering ESBL, KPC, and AmpC-producing strains. Nevertheless, no activity against non-fermenting bacteria has been found [35] (Table 2).

### 7.8. Cefepime/Enmetazobactam

Enmetazobactam rendered cepefime active against CRE including ESBL and KPC strains in a recent in vitro study, but its addition did not increase efficacy against *P. aeruginosa* [35].

Other promising agents whose activity against DTR-GNB in clinical development merit further evaluation include taniborbactam (VNXR-5133), and pyrrolocytosines (RX-P2382), ETX2514, TP-6076, WCK-5153, MEDI3902, COT-143.

## 8. Role of Combination Therapy and Duration of Treatment for DTR GNB BSI

Though the evidence available is low quality (deriving mainly from in vitro and observational studies), empirical and target combination therapy for the treatment of CRE, DTR *P. aeruginosa* and DTR *Acinetobacter* should be considered in the attempt to delay the emergence of antibiotic resistance, especially in the case of severe infections (including BSI) and for critically ill patients. New antibiotics should also be tested in studies, in association with either old antimicrobials (tigecycline, colistin, fosfomycin, old aminoglycosides, carbapenems) and new ones [88,89,90].

Recent studies suggest that short-course (7-day) therapy for GNB BSI may achieve similar clinical response and microbiological cure rates to longer courses (>7 days). However, data are lacking for DTR GNB BSI, and longer treatment duration may be indicated in specific clinical scenarios and/or in BSI due to particular pathogens, especially for non-fermenting GNB. Further studies are urgently needed to test the most promising synergistic combinations and for DTR GNB BSI and to establish the ideal treatment duration [2,91].

## 9. Conclusions

Resistance to currently approved antimicrobials is increasing. The armamentarium against DTR GNB has grown, with the majority of antibiotics active against ESBL and KPC producers. However, there is a need to prioritize MBL producers and DTR *A. baumannii*. Research into the development of new antibiotics showing novel mechanisms of action should be promoted, and the implementation of innovative trial designs should be encouraged, in order to test the new compounds against serious infections, including BSI. In addition, a combination of antibiotics with alternative therapeutic agents for BSI, including adjuvants, bacteriophages, antimicrobial peptides, nanoparticles, and photodynamic light therapy, may open up interesting possibilities for future treatments.

## Figures and Tables

**Table 1:** Classification of most frequent extend ed-spectrum β-lactamases and carpapenemase, spectrum of activity, inhibition, relevant organism and geographic distribution.

Molecular Class	Enzymes	Activity against Penicillins	Activity against Cephalosporins	Activity against Carbapenems	Activity against Aztreonam	Inhibited by Carbapenems	Inhibited by Clavulanic Acid	Inhibited by Tazobactam	Inhibited by Avibactam	Inhibited by Vaborbactam and Relebactam	Relevant Organisms	Geographic Distribution
A (serine penicillinases)	ESBL (TEM, SHV, CTX-M, others)	Yes	Yes, except cefamycins	No	Yes	Yes	Yes	Yes	Yes	Yes	*E. coli* *Klebsiella* spp. *P. mirabilis*	Worldwide spread. Community and nosocomial infections
	KPC	Yes	Yes	Yes Strong activity	Yes	No	No	No	Yes	Yes	*E. coli* *K. pneumoniae* *K. oxytoca* *S. marcescens* *Enterobacter* spp. *C. freundii*	Worldwide United States South and Central America Europe (mainly Italy and Greece)
B (metallo-β-lactamases)	MBLs (VIM, IMP, NDM, others)	Yes	Yes	Yes Strong activity	No	No	No	No	No	Yes	*E. coli* *K. pneumoniae* *K. oxytoca,* *S. marcescens,* *Enterobacter* spp. *C. freundii*	NDM: Asia (mainly India, Pakistan and Bangladesh); Italy (Tuscany) IMP and VIM: Europe (Romania, Poland and Denmark)
C (cephalosporinases)	AmpC type (CMY-2, DHA-1, FOX-1, others)	Yes	Yes except cefepime, including cefamycins	No	Yes	Yes	No	No High concentrations	Yes	Yes	*K. pneumoniae* *E. coli* *Enterobacter* spp. *S. enteritidis* *C. freundii* *S. marcescens* *A.baumannii*	Worldwide spread. Community and nosocomial infections
D (oxacillinases)	OXA (OXA-48, OXA-23, OXA-11, OXA-181, others)	Yes §	Yes Weak activity against cefepime and ceftazidime	Yes Weak activity *	Yes	No	Weak	Weak	Yes	No	*A. baumannii* *P. aeruginosa* *E.coli* *K. pneumoniae* *P. mirabilis* *C. freundii*	Relatively common in Europe (Mediterranean countries)., Northern Africa and Middle Eastern countries Extremely rare in United States

Abbreviation: ESBL, Extended-spectrum β-lactamases; IMP, Imipenemase Metallo-beta-lactamase; KPC, *Klebsiella pneumoniae* carbapenemase; MBLs, metallo-β-lactamases; NDM, New Delhi metallo-betalactamase; OXA-48, oxacillinase-48; OXA-23, oxacillinase-23; VIM, Verona integron-encoded metallo-beta-lactamase. § Except cloxacillin * High-level carbapenem resistance may occur when these enzymes are found in combination with other β-lactamases (ESBL-od Amp-C), or with membrane permeability alteration.

**Table 2:** Spectrum of activity of new antibiotics for difficult-to-treat resistance (DTR) gram-negative bacteria (GNB).

	ESBL	CRE-KPC	CRE-OXA48	CRE-MBL	DTR *P. Aeruginosa*	DTR *Acinetobacter*
**BL/BLI Combination**						
• **Ceftolozane/Tazobactam**	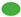	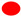	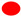	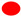	1 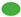	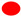
• **Ceftazidime-Avibactam**	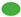	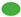	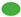	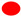	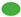	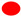
• **Imipenem-Relebactam**	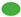	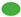	2 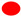	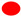	3 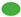	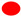
• **Meropenem-Vaborbactam**	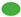	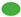	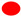	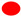	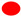	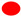
• **Aztreonam-Avibactam**	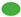	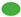	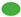	4 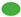	5 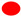	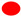
• **Cefepime/Zidebactam**	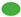	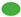	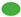	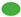	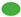	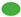
• **Meropenem/Nacubactam**	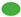	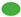	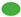	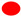	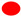	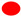
• **Ceftaroline/Avibactam**	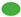	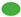	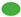	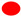	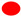	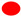
**Novel Cephalosporine**						
• **Cefiderocol**	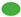	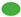	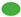	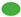	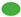	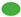
**Novel Amynoglicoside**						
• **Plazomicin**	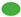	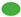	6 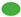	7 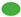	8 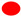	8 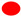
**Novel Tetracycline**						
• **Eravacyclin**	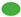	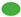	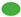	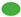	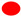	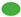
• **Murepavadin**	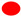	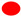	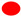	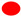	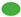	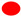

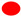
 No activity or intrinsic or acquired resistance. 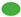 Activity. Abbreviations: BL/BLI, β-lactam/β-lactamase Inhibitor CRE, carbapenem resistant Enterobacteriacae; ESBL, extended-spectrum beta-lactamase; MBLs, metallo-β-lactamases; OMPTA, outer membrane protein targeting antibiotics. 1. Decreased activity for carbapenemase-producing strains of CR *P. aeruginosa*; 2. Very weak activity; 3. Not have activity against MBL; 4. Reduced activity against certain NDM *Escherichia coli* isolates; 5. Activity comparable to aztreonam alone; 6. Activity against OXA-type CREs but increased resistance is observed; 7. Not active against many NDMs; 8. Activity toward *P. aeruginosa* and *A. baumannii* is overall comparable to existing aminoglycosides (tobramycin, amikacin, gentamicin).

**Table 3:** New antibiotics for DTR-GNB currently approved with dosing, pros and cons.

Drug Name	Dosing for Patients with *Normal Kidney Function*	Comments
**Ceftolozane-tazobactam**	1.5 (ceftolozane 1 g + tazobactam 0.5 g) IV q 8 h iv for UTI and IAI 3 g (ceftolozane 2 g + tazobactam 1 g) IV q 8 h for NP	PROS: rapid tissue distribution (including lung); safe; limited activity also against *S.maltophilia* CONS: no activity against most anaerobes, staphylococci and enterococci; variably susceptibility of AmpC producers, two dosages, lower success in patients with CRRT
**Ceftazidime-avibactam**	2/0.5 g IV q 8 h	PROS: rapid tissue distribution (including lung); safe; approved for empiric and targeted therapy of DTR GNB; combination treatment with aztreonam against MBL CONS: no activity against most anaerobes staphylococci and enterococci; lower success in patients with CRRT; resistance
**Imipenem-relebactam**	1.25 g (imipenem 500/cilastatin 500/relebactam 250 mg) IV q 6 h	PROS: possible activity in strains resistant to both ceftolozane/tazobactam and ceftazidime/avibactam CONS: risk of neurotoxicity
**Meropenem-vaborbactam**	2/2 g IV q 8 h	PROS: promising efficacy in the setting of pneumonia and/or other severe KPC -CRE infections; resistance selection overall lower than ceftazidime/avibactam CONS: not active against Amber class B or D carbapenemases
**Cefiderocol**	2 g IV q 8 h (2 g IV q 6 h if CrCl > 120 mL/min)	PROS: novel siderophore cephalosporin; promising for the treatment of DTR *A. baumannii* no activity also against *S. maltophilia* CONS: not activity against gram positives and anaerobes
**Plazomicin**	15 mg/kg IV q 24 h	PROS: once daily dose; carbapenem or β-lactam/β-lactamase inhibitor sparing alternative for UTI CONS: no activity against anaerobes, *A. baumannii, S. maltophilia*,; lower risk for aminoglycosides associated toxicity
**Eravacycline**	1 mg/kg IV/oral q 12 h	PROS: oral formulation, activity also against gram positive (including MRSA, VRE) bacteria, anaerobes, *S. maltophilia* CONS: suboptimal urinary pharmacokinetics; limited data in pneumonia and BSI; no activity against *P. aeruginosa*

BSI, blood stream infections; CRE; carbapenem resistant *Enterobacteriales*; CRRT, continuous renal replacement therapy; DRT, difficult to treat resistance; GNB, Gram negative bacteria; IAI, intrabdominal infections; KPC, *Klebsiella pneumoniae* carbapenemase producers; MBL, metallobetalactamase; MRSA, *methicillin*-*resistant* *Staphylococcus aureus*; NP, nosocomial pneumonia; UTI, urinary tract infections; VRE, vancomycin-resistant *Enterococcus*.

## Abbreviations

BL/BLI	β-lactam/β-lactamase inhibitor
BSI	blood stream infections
CRE	carbapenem-resistant *Enterobacteriales*
CAZ/AVI	ceftazidime/avibactam
C/T	ceftolozane/tazobactam
cIAI	complicated intra-abdominal infections
cUTI	complicated urinary tract infections
DTR	difficult-to-treat resistance
ESBL	Extended-spectrum Beta-lactamase
GNB	Gram-negative bacteria
HAP	hospital acquired pneumonia
IMP	imipenemase
KPC	*Klebsiella pneumoniae* carbapenemases
MBL	metallo β-lactamases
MDR	multidrug resistant
NDM	new Delhi metallo beta-lactamase
PDR	pandrug resistant
NP	nosocomial pneumonia
OXA	oxacillinases
VAP	Ventilator associated pneumonia
VIM	Verona integron-borne metallo-β-lactamase
XDR	extensive drug resistance
